# *Yersinia pestis* halotolerance illuminates plague reservoirs

**DOI:** 10.1038/srep40022

**Published:** 2017-01-05

**Authors:** Maliya Alia Malek, Idir Bitam, Anthony Levasseur, Jérôme Terras, Jean Gaudart, Said Azza, Christophe Flaudrops, Catherine Robert, Didier Raoult, Michel Drancourt

**Affiliations:** 1 Aix Marseille Université, URMITE, UMR 63, CNRS 7278, IRD 198, Inserm 1095, Faculté de Médecine, 27 Bd Jean MOULIN, 13385 Marseille Cedex 5, France; 2 Laboratoire Biodiversité et Environnement: Interactions Génomes, Faculté des Sciences Biologiques Université des Sciences et de la Technologie Houari Boumediene, El Alia, Bab Ezzouar 16111, Algérie; 3 Aix-Marseille Université, UMR912 SESSTIM (INSERM/IRD/AMU), Faculté de Médecine, 27 Bd Jean Moulin, 13385 Marseille Cedex 5, France

## Abstract

The plague agent *Yersinia pestis* persists for years in the soil. Two millennia after swiping over Europe and North Africa, plague established permanent foci in North Africa but not in neighboring Europe. Mapping human plague foci reported in North Africa for 70 years indicated a significant location at <3 kilometers from the Mediterranean seashore or the edge of salted lakes named chotts. In Algeria, culturing 352 environmental specimens naturally containing 0.5 to 70 g/L NaCl yielded one *Y. pestis* Orientalis biotype isolate in a 40 g/L NaCl chott soil specimen. Core genome SNP analysis placed this isolate within the *Y. pestis* branch 1, Orientalis biovar. Culturing *Y. pestis* in broth steadily enriched in NaCl indicated survival up to 150 g/L NaCl as L-form variants exhibiting a distinctive matrix assisted laser desorption-ionization time-of-flight mass spectrometry peptide profile. Further transcriptomic analyses found the upregulation of several outer-membrane proteins including TolC efflux pump and OmpF porin implied in osmotic pressure regulation. Salt tolerance of *Y. pestis* L-form may play a role in the maintenance of natural plague foci in North Africa and beyond, as these geographical correlations could be extended to 31 plague foci in the northern hemisphere (from 15°N to 50°N).

Plague is a deadly infectious disease caused by the bacterium *Yersinia pestis*^1^,^2^. Post-genomic analyses confirmed that *Y. pestis* was derived from the environmental bacteria *Yersinia pseudotuberculosis* 3,000 to 6,000 years ago in Central Asia and gradually spread from east to west along the historical tracks of human migration such as the Silk Road^3^,^4^,^5^,^6^,^7^. *Y. pestis* reached Europe and North Africa where it caused a pandemic called the Justinian pandemic between 541–767 AD; then a medieval pandemic between 1346 and the end of the eighteenth century^8^,^9^. These two historical pandemics, characterized by an explosive mortality, killing up to half of the urban populations in a few months, have been microbiologically confirmed by the detection of specific nucleotidic sequences^10^,^11^,^12^ and the reconstitution of the entire genome of several strains^5^,^13^,^14^,^15^. This epidemic regimen, which has never been observed thereafter, was likely fueled by inter-human transmission by human ectoparasites such as lice^16^,^17^,^18^,^19^,^20^,^21^ and the *Pulex irritans* fleas^22^. A third pandemic began in the Hong Kong area in the late nineteenth century^2^. It is currently responsible for hundreds of deaths every year. It exhibits a different epidemiological regimen characterized by epidemics circumscribed in time and in more limited geographical areas that are called plague foci^23^. These epidemics are linked to transmission of *Y. pestis* by small wild mammal ectoparasites without inter-human transmission^1^. Moreover, small outbreaks are linked with contact with infected cats^24^ or consumption of infected meat^25^ and sporadic cases described at the southern border of the United States are due to direct contact with infected wild animal carcasses^26^.

Strikingly, two millennia after swiping over Europe and North Africa, plague established foci in later regions but not in neighboring Europe^4^. In North Africa, plague foci are still active as illustrated by the resurgence of the plague after 53 years of silence in Oran, Algeria, for which genetic analyses confirmed a local and not imported strain of *Y. pestis*^27^. In these foci, plague is a zoonosis since the *Y. pestis* bacterium is introduced into populations from infected animals^1^. These constitute a link in an epidemiological chain involving a balanced transmission between plague-susceptible and plague-resistant species^28^.

It is likely that, ultimately, animals become contaminated from infected soil^29^,^30^. Indeed, *Y. pestis* was isolated from rodent burrow soil several years after any animal had actually lived there^31^. Also in natural conditions, a strain of *Y. pestis* was isolated from the ground at the point of death of a mountain lion with plague, three weeks after the death of the animal^26^. Experimental data have confirmed several times the persistence of living *Y. pestis* up to 28 months after artificial inoculation of soil^32^,^33^.

The reasons for the persistence of plague foci in North Africa and not in neighboring Europe are not understood. In this context, we observed that in North Africa plague foci were significantly located at the periphery of chotts, which are salty areas with a salt content from 10 g/L to saturation (300–400 g/L), higher than that of the seas and oceans^34^. We isolated a new strain of *Y. pestis* in Algeria in a chott soil sample containing 40 g/L of salt. Finally, we showed that the persistence of *Y. pestis* in soil samples artificially inoculated with this strain was the same in the presence of salt, but as L-form like variants that had been poorly described for this bacterial species.

## Results

### Co-localization of plague foci and chotts, North Africa

**Figure 1:**
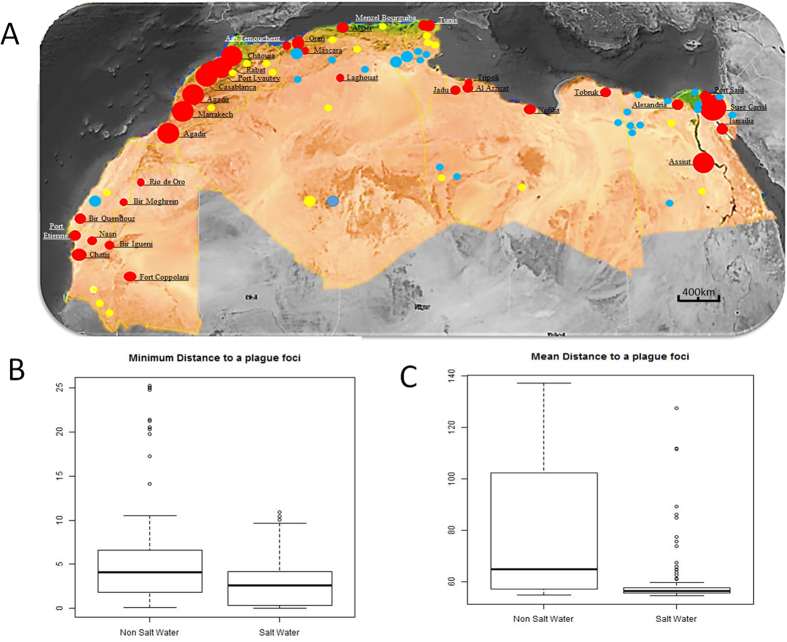
(**A**) Location of human plague foci in six countries in North Africa, 1940–2015. Plague foci are significantly located <3 km of salt source (Mediterranean sea and chotts). This figure was generated from the map of the software Google Maps/Google Earth and Google Maps/Google Earth APIs (https://www.google.com/permissions/geoguidelines.html). 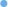
Salt water, 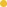
Fresh water; 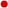
1–10 cases; 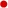
10–100 cases; 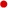
>100 cases. (**B**) Boxplot of minimum distances to plague foci (y axis, distance in km). (**C**) Boxplot of mean distances to plague foci (y axis, distance in km).

In North Africa, plague reemerged in Oran, Algeria, in 2003^27^, 53 years after a previous episode in the same city. Indeed, this was an intriguing reminiscence of the famous Nobel-prized Albert Camus book (The Plague) situated in Oran. Another reemergence took place in Tobruk, Libya, in 2009^35^,^36^. Both outbreaks are located on the edge of the Mediterranean Sea with a 30–35 g/L salinity in addition to the edge of the Sebkha with up to 400 g/L salinity for the Algerian cases. We mapped the human plague foci reported in North Africa for 75 years including the exhaustive work by M. Baltazard in Morocco and observed that these foci were all located at a distance <3 kilometers from the sea or from the edge of a chott, which designates an inland salty area (Fig. 1). Salt water ponds were significantly closer to plague foci than non-salt water ponds, according to the minimum distance (Fig. 1) (Median [IQR] 2.67 km [3.76] vs 4.12 km [4.68], p < 0.001). Salt water ponds were also significantly closer in mean to plague foci (Median [IQR] 56.30 km [1.87] vs 64.83 km [44.22], p < 0.001) (Fig. 1). Furthermore, we measured a significant spatial association between human plague for 31 foci and saline water in comparison to freshwater on the northern hemisphere (from 15°N to 50°N) (Supplementary Fig. 1). Significant statistical analysis showed that the median distance to a plague focus is greater for freshwater sources than for salt springs. Three quarters of the salt springs are located less than three km away while three quarters of freshwater sources are further than three km away. Indeed, the median distance of a plague focus to a salt water point is 0.89 (IQR ± 2.66) with a minimum of 0.01 km and a maximum of 7.74 km, while the median distance to a freshwater source is 4.63 (IQR ± 2.7) km with a minimum of 0.09 km and a maximum of 9.95 km.

### Isolation of *Y. pestis* in chott

**Figure 2:**
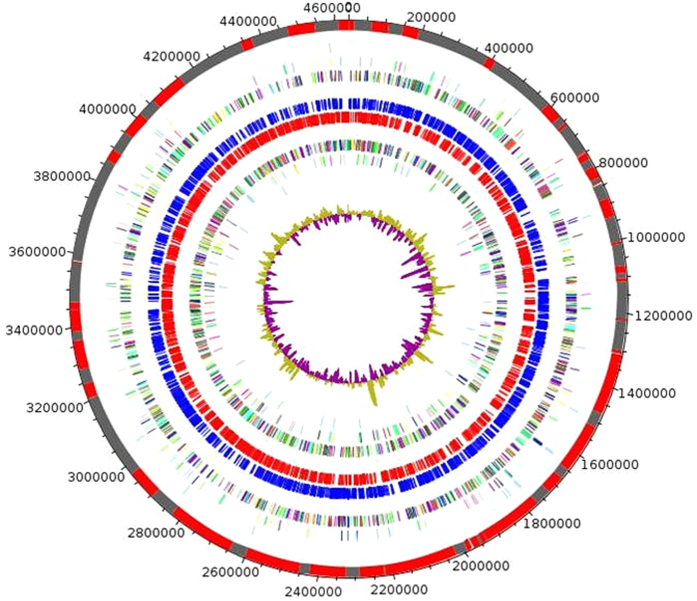
Circular genome map of *Y. pestis* Algeria3. From outside to center: Contigs (red/grey), COGs category of genes on forward strand (three circles), genes on forward strand (blue circle), genes on reverse strand (red circle), COGs category on reverse strand (three circles), G + C content. COGs, Clusters of Orthologous Groups database.

**Figure 3:**
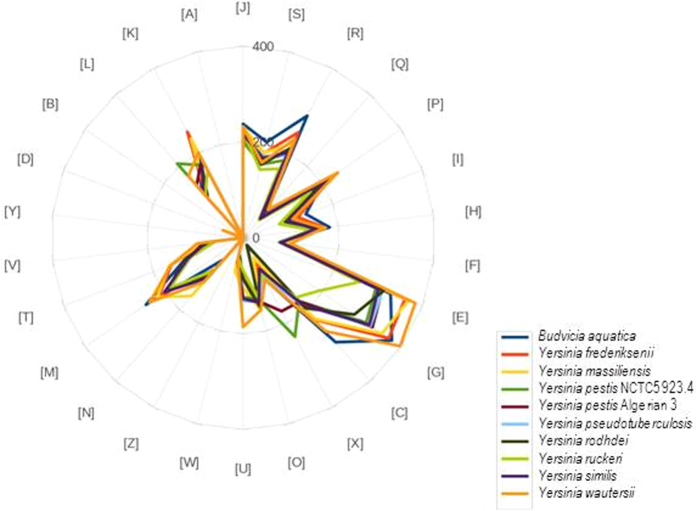
Distribution of functional classes of predicted genes according to the clusters of orthologous groups of proteins.

**Figure 4:**
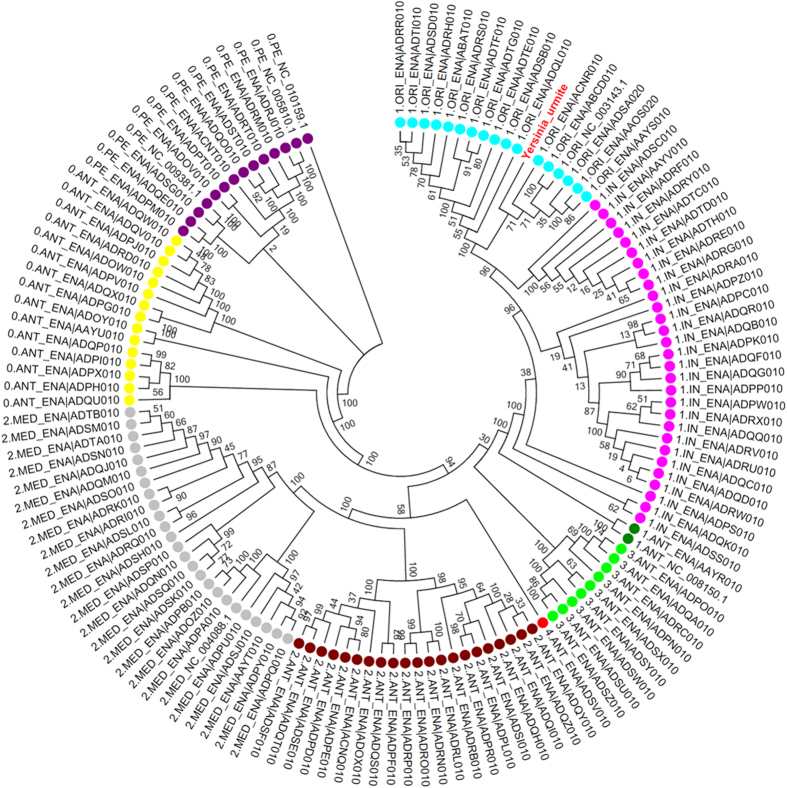
Phylogenetic tree of 134 *Y. pestis* genomes based on the 2,298 SNPs. Biovar were designated as follows: ORI (Orientalis), ANT (Antiqua), In (intermediate strains between ANT and ORI), MED (Medievalis) and PE (Pestoides). Each strain was referred as branch 1, 2, 3 or 4 according to Cui *et al*.^6^.

To investigate the presence of *Y. pestis* in chotts in Algeria, we collected 208 soil samples comprising: 120 soil samples collected in chotts (salinity, 25 to 70 g/L) and 88 soil samples collected outside chotts (salinity, 0.5 to 5 g/L); and 144 water samples of which 98 contained salt water (50 g/L NaCl) and 46 contained freshwater (0.05 to 5 g/L NaCl) (Supplementary Table 1). Culturing these 352 samples in a safety level 3 laboratory yielded one *Y. pestis* isolate (named *Y. pestis* Algeria3) in one chott soil specimen containing 4% NaCl against zero isolates from low salinity control soil samples. The genome of *Y. pestis* Algeria3 (GenBank accession number FAUR00000000) (Fig. 2) is 4,637 Mbp-long and composed of 48 scaffolds with a GC content of 47.65%. Of the 4,203 predicted genes, 4,115 were protein-coding genes (Table 1). Eighty-eight RNAs were detected including seven 5S rRNA, six 16S rRNA, five 23S rRNA and 70 tRNA genes. A total of 3,455 genes (83.9%) were assigned as putative function and eight genes were identified as ORFans. The distribution of genes into COGs functional categories is presented in Supplementary Table 2. The global distribution of genes into COGs categories was comparable across all compared genomes including *Yersinia pestis*_NCTC_5923,4, *Yersinia similis, Yersinia frederiksenii, Yersinia rodhdei, Yersinia ruckeri, Yersinia massiliensis, Yersinia wautersii, Budvicia aquatica, Yersinia pseudotuberculosis* (Fig. 3). When *Y. pestis* Algeria3 was compared to other *Yersinia* species, the average percentage similarity of nucleotides corresponding to orthologous protein shared between genomes ranged from 64% for *Y. rohdei* to 94% for *Y. wautersii* (Supplementary Table 3). Based on 16S molecular marker, the phylogenetic position of *Y. pestis* Algerian3 placed this strain within the *Y. pestis* clade. We also compared *Y. pestis* Algeria3 with other *Y. pestis* strains using DNA-DNA hybridization (DDH) (Supplementary Table 4) and confirmed that *Y. pestis* Algeria3 isolate was biotyped Orientalis *Y. pestis*. Finally, the analysis of 2,298 SNPs on a collection of 133 genomes in addition to *Y. pestis* Algeria3 genome, allowed to reconstruct the complete phylogeny of *Y. pestis*. This comparative analysis disclosed that *Y. pestis* Algeria3 belongs to the branch 1of *Y. pestis* and clusterized with the Orientalis biovar strains (Fig. 4)^6^. In order to confirm this observation, we compared the survival of *Y. pestis* Algeria1, another Orientalis isolate from Algeria^37^, after its experimental inoculation in three different natural soils collected in Algeria, sterilized by autoclaving and supplemented with 0.5 g/L NaCl or 40 g/L NaCl. There was no difference in the 5-week survival of *Y. pestis* Algeria1 in these two seeded soil conditions (p = 0.15) (Supplementary Table 5). Survival in salted soil was not restricted to just the Algeria3 isolate.

### Salt-induced *Y. pestis* L-forms

**Figure 5:**
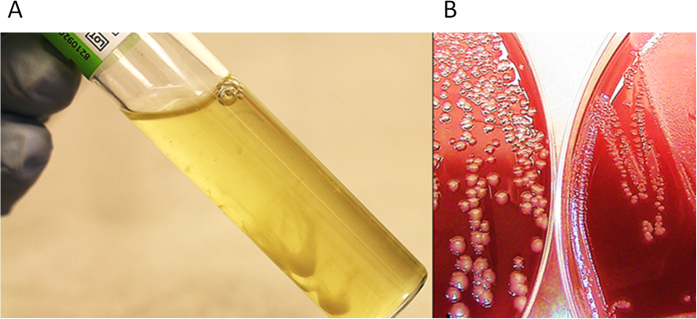
*Yersinia pestis* forms filamentous colonies in 150 g/L NaCl-broth (**A**) and small colonies (**B**): left panel, control; right panel, *Y. pestis* exposed to 150 g/L NaCl.

**Figure 6:**
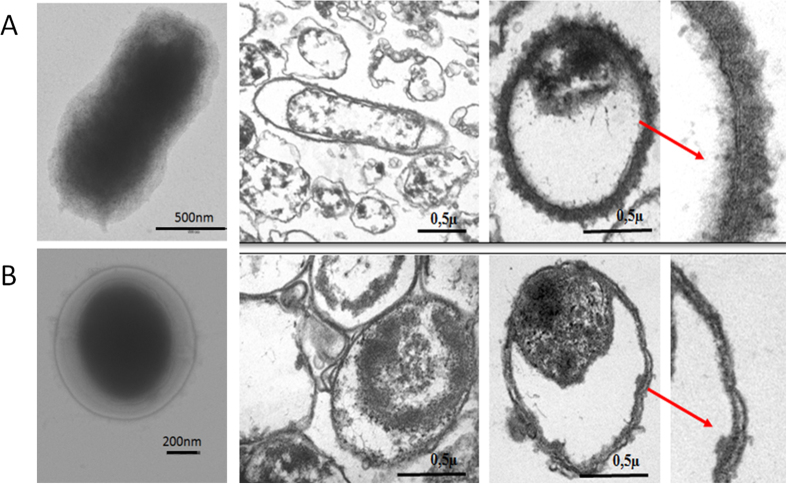
Electron microscopy of *Y. pestis* control (**A**) and *Y. pestis* exposed for seven weeks to 150 g/L NaCl (**B**). Red arrows point to enlarged cell wall, thicker in control cells (**A**) than in *Y. pestis* cells exposed to 150 g/L NaCl (**B**).

To further characterize *Y. pestis* cells exposed to salt, we cultured *Y. pestis* Algeria1 in trypticase-soy broth supplemented with an increasing concentration of salt in the presence of a control cultured in parallel in standard broth. After a seven-week culture in broth containing 150 g/L NaCl, *Y. pestis* formed large filamentous colonies that troubled the broth (Fig. 5A). Subculture of these colonies on 5% sheep-blood agar yielded opaque white microcolonies with a smooth surface, a regular round shape and an entire edge like the control strain but smaller (0.9–1 mm) (Fig. 5B). Such colonies were less sticky and easier to take off completely losing their natural viscosity than the control *Y. pestis* cell colonies, which formed sticky strands when touched by an inoculation loop. The colony became whiter to opaque when we poured trypticase-soy broth on the Petri dish. Gram staining disclosed morphological changes of *Y. pestis* for salt concentrations starting at 50 g/L NaCl; at lower NaCl concentrations, *Y. pestis* showed a classic morphology featuring individual coccobacilli whereas for concentrations above 50 g/L NaCl, *Y. pestis* featured aggregate cocci of a small size. Negative staining and electron microscopy showed the control culture to contain bacilli measuring 1,340 ± 72 μm by 810 ± 21 μm (Fig. 6) whereas *Y. pestis* exposed to 150 g/L NaCl exhibited a round shape and measured 973 ± 32 μm by 901 ± 25 μm (P < 0.05). Inclusion electron microscopy showed that, compared to control *Y. pestis* cells, cells of *Y. pestis* exposed to 150 g/L NaCl exhibited a thinner cell membrane and a thinner inner membrane, which eventually vanished as reported in spheroplasts (L-forms)^38^ (Fig. 6).

### Salinity induces a specific transcriptional program in L-form *Y. pestis*

**Figure 7:**
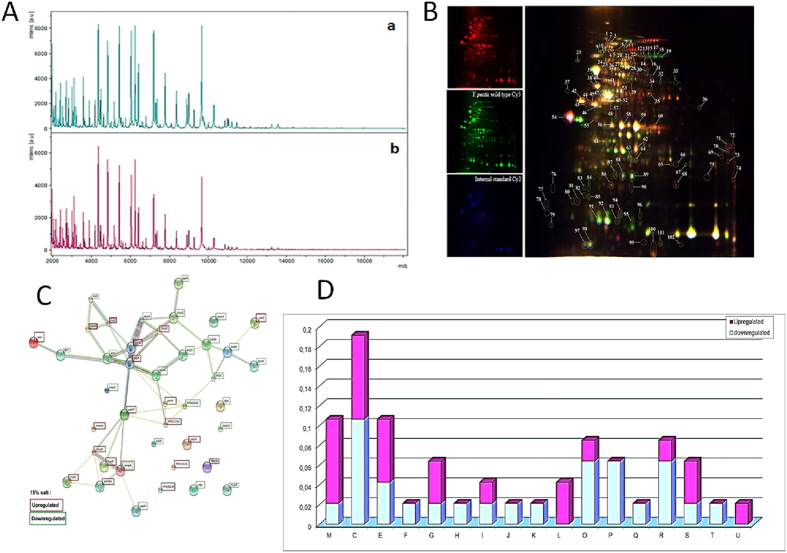
Proteomics of *Y. pestis* exposed to 150 g/L NaCl: (**A**): MALDI-TOF mass spectrometry (a: control; b: exposed) (**B**). Representative 2D differential gel electrophoresis (DIGE) analysis of *Y. pestis* proteins (**C**): string network of DIGE analysis of *Y. pestis* proteins. Each individual sample from *Y. pestis* Orientalis wild type and 150 g/L NaCl exposed *Y. pestis* and a pooled reference sample were labeled with Cy5, Cy3, and Cy2, respectively, and were then separated on the same gel using the 2D-DIGE system. Three images were obtained from each gel and an overlay of dye scan images was also obtained. Selected protein spots exhibiting an ANOVA score lower or close to 0.05 and a change of at least 1.5-fold intensity are indicated by circles and spot numbers as indicated in Table (**D**): Analysis, according the COG family, of upregulated and downregulated proteins identified by mass spectrometry after 2D-differential gel electrophoresis separation.

The analysis of protein spectra obtained by MALDI-TOF-MS spectrometry confirmed the identification of *Y. pestis* Algeria1 with an identification score of 2.299–2.5; and revealed a reproducible pattern with the same distribution but with slight differences in absolute intensity between those obtained with the reference strain (Fig. 7). Furthermore, of the 102 spots obtained by 2D differential gel electrophoresis separation, 68 were identified by MS analysis which yielded 42 proteins belonging to 17 different COGs. COG M, C and E contained the majority of the differentially regulated proteins. More particularly, upregulation was observed for: outer-membrane proteins OmpF, OmpA and TolC (hasF), an outer-membrane efflux pump; increased energy production; as well as for L-lactate dehydrogenase; proteins involved in cellular processes and signaling such as YaeT; proteins involved in metabolism such as lldD, nuoD, atpD, atpA, aspA, proV, ureC, ppSA, lamB and fadL; in information storage and processing such as ssb and RarA; and finally other proteins poorly characterized. On the other hand, the D-lactate dehydrogenase, dihydrolipoamide dehydrogenase, beta succinyl-CoA synthetase subunit and catalase katE and katY were downregulated.

## Discussion

We present several lines of evidence that the plague agent *Y. pestis* is surviving specifically in salt soil environments such as the ones encountered in so-called chotts in North Africa. Indeed, after we observed significant co-localization of plague foci reported for 70 years in animals and humans in North Africa, with salt chotts, we cultured one *Y. pestis* Algeria3 isolate from one chott soil specimen containing 40 g/L NaCl, whereas no isolate was produced from control soil specimens containing 0.5–1 g/L salt. Algeria3 is the first soil isolate of *Y. pestis* in Africa, as the three previous isolates made over 120 years were in Asia^2^,^31^ and America^26^. Experimental studies further confirmed that *Y. pestis* survived in soil containing 40 g/L salt and in hypertonic broth containing up to 150 g/L salt. Interestingly, beside *Vibrio* spp. and *Halomonas* spp. organisms, very few halotolerant bacteria are pathogenic^39^,^40^.

*Y. pestis* exposed to salt formed round cells looking like L-forms. More than one century ago, “involution forms” were reported after exposure of *Y. pestis* to 35 g/L NaCl^41^,^42^. “Involution forms” are convincingly identical to L-forms photographed after culture of *Y. pestis* in 20 g/L salt broth^43^. Interestingly, several Russian reports made in the 70–80 s described *Y. pestis* L-forms in rodents and their ectoparasites collected in plague foci^44^.

*Y. pestis* did not sustain direct exposure to high NaCl concentrations, but rather a progressive, stepwise exposure to high salinity. This observation suggests either the selection of halotolerant variants within the population or, more likely, a progressive adaptation to increasing NaCl concentrations. L-forms may reflect an adaptation of *Y. pestis* to osmotic pressure by decreasing the surface in contact with the environment. In line with these morphological observations, we noticed upregulation of several outer-membrane proteins including efflux pumps such as TolC (linked to the inner membrane AcrB), exporting toxic compounds to the bacteria^45^, and OmpF porin regulating the osmotic pressure to maintain cell permeability^46^. Whereas *Escherichia coli* OmpF is upregulated by low osmolarity and repressed by high osmolarity, *Y. pestis* OmpF expression is not repressed by high osmolarity but is incredibly upregulated^46^. Alternatively, halotolerance in *Y. pestis* may rely on the Na^+^/H^+^ antiporter (nhaA and nhaB), recently described as necessary for the virulence of *Y. pestis*^47^. It has been shown that the Na^+^/H^+^ antiporters in the model of *Vibrio cholerae*, the causative agent of cholera, confer Na^+^ resistance involving the NADH-quinone oxidoreductase pump (NQR)^48^.

Extending our geographic investigations in North Africa to the north hemisphere, we observed that plague foci are indeed significantly located close to salt sources (Supplementary Fig. 1). This observation is no longer true for the south hemisphere, where the majority of human cases are reported today. Accordingly, several metallic ions including calcium, magnesium, iron and selenium have been suspected to play a role in the maintenance of *Y. pestis* in plague foci^49^,^50^.

We conclude that salt is one of the factors contributing to the maintenance of plague foci in North Africa and Eurasia. L-form of *Y. pestis* may persist in these salt areas. Indeed, salinity may modulate plant and rodent populations and density and play an indirect role in plague foci. Based on maps of salt in the north hemisphere, it may be possible to focus the surveillance of enzootic plague around salt sources in order to optimize the prevention of human plague in these countries.

## Materials and Methods

### Analyzing co-localization of plague foci and chotts

In a first step, chotts were localized in the five North African countries Mauritania, Morocco, Algeria, Tunisia and Lybia by using the Map Atlas on line (http://www.unesco.org/languages-atlas/fr/atlasmap.html). Then plague outbreaks reported in these five countries over 75 years were plotted. Coordinates corresponding to the middle of every focus were used for statistical analysis. Distance between each water pond and plague foci was estimated according to geographical coordinates. For each water pond (either salt or non-salt water), the shortest distance (minimum distance to plague foci) and the mean distance were estimated. Salt and non-salt water categories were compared using a non-parametric Wilcoxon test. Statistical analyses were performed using R3.1.3 software (Copyright 2015 The R Foundation for Statistical Computing, Vienna, Austria). In a second step, this analysis was extended to the northern hemisphere in areas from 15°N to 50°N. For the study we considered plague foci described in the last 50 years by the World Health Organization (WHO) as well as human cases reported and sylvatic plague, because in some countries no human cases do not mean no plague.

### Sampling on an inanimate environment

Based on the assumption of co-localization of plague foci and a salty environment, we conducted a field mission for sampling soil and water in plague foci in Algeria. We investigated Algerian plague foci described during the 2000 s: in Oran^27^ from the Sebkha (Aïn Beida side, Daët el Bagrat and El Kerma side), Oued Chelif, Daiat Morsly, Gharabas lake, Telamine lake and from Saline of Arzew; in Laghouat^51^ from M’Zi oued, whose courses end up in Chott El Melghir; in M’Sila^52^ from Chott el Hodna (Maarif side, Souamaa side and Ain el Khadra); and in Biskra^48^ from Oumache, Ourelal, Bouchegroune and Tolga. Salinity was tested using a refractory technique (automatic salinity refractometer, Fisher Scientific, Strasbourg, France).

### *Yersinia pestis* strains

*Y. pestis* Algeria1 strain CSUR (Collection de Souches de l’Unité des Rickettsies) P100, an Orientalis biotype isolate made from a rodent in Algeria^37^, was used in this study. In addition, a *Y. pestis* Algeria3 isolate was made directly from one natural soil specimen collected at Chott el Hodna, Algeria (Supplementary Table 1). Manipulations of *Y. pestis* were performed in a biosafety level 3 laboratory. *Yersinia* cells were streaked from a frozen culture stock onto 5% sheep-blood agar (bioMérieux, La Balme-les-Grottes, France) for 48 hours at 28 °C. As for the experiment, *Yersinia* cells were grown in two tubes, each containing 10 mL trypcase soy broth (0.5% of sodium chloride in this medium; Becton-Dickinson, Grenoble, France) to the early stationary growth phase at 10^8^ colony-forming units (CFU)/mL (OD600 = 2; Biolog model 21907S/N 0744984). One control tube and the second tube supplemented with 10 g/L enzyme and endotoxin-free NaCl (Carlo Erba, Mundolsheim, France) were incubated at 28 °C for 5 days. Then, *Yersinia* cells were subcultured on 5% sheep-blood agar (without NaCl addition) (ref 43041, bioMérieux, Marcy l’Etoile, France) to check for cell viability and enumerate the colonies. Colony enumeration was done after the inoculum had been serially diluted 1:10 to 10E-6 dilution and digital pictures of colonies were recorded. An average was calculated for dilutions up to about 10 to 300 colonies. The counting CFU/mL is expressed in log10. Also, acridine orange (Becton-Dickinson, Grenoble, France) staining was performed. Slides were washed, mounted with Fluoprep (bioMérieux) and examined using an optical microscope (x1,000) (Leica, Saint-Jorioz, France) and under the Leica DM2500 Upright Fluorescence Microscope at x1,000 magnification. The identification of all colonies was confirmed using matrix-assisted laser desorption/ionization time-of-flight mass spectrometry (MALDI-TOF-MS) and a Microflex system (Brücker Daltonics, Wissembourg, France) as previously described^53^. In parallel, 5 mL of the inoculated salt-culture medium were mixed with 5 mL of a salt-trypcase soy broth solution to increase the concentration at 2% w/v NaCl. The remaining volume was kept to verify the viability of cells under these conditions by subcultures every 48 hours. This operation was repeated up to a final NaCl concentration of 15% w/v NaCl (150 g/L). Also, *Y. pestis* cells were directly subcultured into trypticase-soy broth containing either 5% w/v, 10% w/v or 15% w/v NaCl final concentration, incubated for 7 days and subcultured on sheep-blood agar every 48 hours. DNA was extracted from the strain using the QIAamp DNA Mini Kit (Qiagen) according to the manufacturer’s protocol. PCR sequencing of partial *yopT, caf1, ymT* and *pla Y. pestis* genes was carried out in an Applied Biosystems 2720 thermal cycler (MJ Research) to demonstrate that the strain had kept its three plasmids: pFRa, pPla and pYV. Experiments were done in triplicate.

### Electron microscopy

Control *Y. pestis* cells subcultured without NaCl and *Y. pestis* cells exposed to 150 g/L NaCl for 7 weeks were examined by electron microscopy using both negative staining and inclusion. Observations were made using a Morgagni 268D microscope (FEI, Philips, France) operating at X35-X280000 magnification. Negative stains were made by contrasting samples with a solution of 1% ammonium molbydate, inclusions were performed after sample fixation in a 2% glutaraldehyde. For each grid, a minimum of 150 *Y. pestis* cells were observed, and a minimum of five grids were observed for each condition.

### *Yersinia pestis* genome sequencing

Genomic DNAs of the of *Y. pestis* Algeria3 strain was sequenced on the MiSeq Technology (Illumina, Inc, San Diego CA 92121, USA) with paired end strategy. One ng of each genomic DNA was tagmented, indexed and normalized according to the Nextera XT library kit (Illumina).

Automated cluster generation and paired-end sequencing with dual index reads was performed on two 2 × 251-bp runs. Total information of 4.6 Gb was obtained from these runs, with 8,819,290 clusters passing quality control filters. Index representation of *Y. pestis* attributed 459,092 paired reads to *Y. pestis* Algeria3 used for the assembly. Open reading frames (ORFs) were predicted using Prodigal^54^ with default parameters. The predicted ORFs were excluded if they spanned a sequencing gap region (containing N). The predicted bacterial protein sequences were searched against the GenBank database and the Clusters of Orthologous Groups (COGs) database using BLASTP (*E* value 1e^−03^, coverage 0.7 and 30% identity). If no hit was found, search was then done against the NR database using BLASTP with an *E* value of 1e^−03^, coverage 0.7 and 30% identity. The tRNAs and rRNAs were predicted using the tRNA Scan-SE and RNAmmer tools, respectively^55^,^56^. SignalP and TMHMM were used to screen the signal peptides and the number of transmembrane helices, respectively^57^,^58^. For each selected genome, complete genome sequence, proteome genome sequence and Orfeome genome sequence were retrieved from the FTP site of National Center for Biotechnology Information (NCBI). All proteomes were analyzed using proteinOrtho^59^. An annotation of the entire proteome was performed to define the distribution of functional classes of predicted genes according to the clusters of orthologous groups of proteins. For phylogenetic tree construction, sequences were recovered by nucleotide blast against the 16S RNA Database of “The All-Species Living Tree” Project of Silva. Sequences were aligned using Muscle and phylogenetic inferences obtained using the approximately-maximum-likelihood method within the FastTree software^60^. Numbers at the nodes are support local values computed with the Shimodaira-Hasegawa test. For core genome SNPs analysis, all 133 genomes analyzed by Cui *et al*. along with the one of *Y. pestis* Algeria3 were downloaded from NCBI or European Bioinformatics Institute (EMBL-EBI). Based on 2,298 SNPs, we used the maximum likelihood method (ML) for the phylogenetic reconstruction. Bootstrap analysis was then undertaken using 100 repetitions.

### 2D-DIGE analysis

We performed the Two-Dimensional (2D) Difference Gel Electrophoresis technique for separating complex mixtures of proteins for *Y. pestis* control and *Y. pestis* at the maximum level of salinity to visualize the effect of salt on proteome. The four replicates of each strain condition were prepared as in the standard 2D-PAGE using 2-D Clean-Up Kit.

Sample preparation for 2D-DIGE: purified bacteria were resuspended in solubilization buffer (30 mM Tris, 7 M urea, 2 M thiourea and 4% (w/v) 3-[(3 cholamidopropyl)-dimethylammonio]-1-propanesulfonate (CHAPS) and disrupted by sonication (three times for 60 s at power = 20 W without pulsing). Cell debris was removed by centrifugation (12,000 × g, 4 °C, 10 min), and soluble proteins were precipitated using the Plus One 2-DClean-Up Kit (GE Healthcare, Chalfont St. Giles, UK). The final pellet was resuspended in solubilization buffer. The protein concentration was determined using the Bio-Rad protein assay kit (Bio-Rad). After this step, the pH of the sample was adjusted if needed to 8.5 (the appropriate pH for sample labeling). For 2D-DIGE analysis and protein identification, four replicates from each medium were labeled with cyanine dyes (Cy3 or Cy5) in a ratio of 400 pmol CyDye to 50 μg of protein, according to the manufacturer’s instructions (GE Healthcare, Chalfont St Gilles, UK). An internal standard was created by combining equal amounts of protein from every sample and then labeling with Cy2 using the same ratio. Each sample was labeled for 30 min on ice in the dark and the reaction was quenched by the addition of 1 μl of 10 mM lysine. CyDye-labeled samples were combined during 2-D gel electrophoresis so that each gel contained a Cy2, a Cy3 and a Cy5 labeled sample. Two dimensional gel electrophoresis was carried out as previously described^61^. After electrophoresis, gels were scanned at appropriate wavelengths to cyanines using the Typhoon FLA 9000 Imager according to the manufacturer’s protocol (GE Healthcare). Scans were acquired at 100 μm resolution. Images were cropped with Image Quant TM software (GE Healthcare) and further analyzed using the Progenesis SameSpots software version 4.0.3779 from Nonlinear Dynamics (Newcastle upon Tyne, UK) as described by the manufacturers. To determine significant differences in 2D spot abundance, an ANNOVA score (p-value) lower or close to 0.05 and a change of at least 1.5-fold between the wild type *Y. pestis* and the salt stress *Y. pestis* protein spots were required for spots to be selected for digestion and identification by MS analysis as previously described^61^.

### MS protein identification

After protein fractionation and relative quantity measurement by 2D-DIGE, all proteins from gel spots were reduced then alkylated and finally in-gel digested. The MALDI-TOF-MS spectra peaks lists were compared to *Y. pestis* protein sequences with our internal Mascot search engine (IHU).

## Additional Information

**How to cite this article**: Malek, M. A. *et al. Yersinia pestis* halotolerance illuminates plague reservoirs. *Sci. Rep.* **7**, 40022; doi: 10.1038/srep40022 (2017).

**Publisher's note:** Springer Nature remains neutral with regard to jurisdictional claims in published maps and institutional affiliations.

## Supplementary Material

Supplementary Information

## Figures and Tables

**Table 1:** Properties of the *Y. pestis* Algeria3 genome.

	Total
Size (bp)	4,637,400
GC content	47.65%
Coding region (bp)	3,881,397
Total genes	4,203
RNA genes	88
Protein-coding genes	4,115
Genes with function prediction	3,455
Genes assigned to COGs	2,996
Genes with peptide signal	727
