# Supplementary material for: Yersinia pestis halotolerance illuminates plague reservoirs
Source: Sci Rep. 2017 Jan 5;7:40022. doi: 10.1038/srep40022 (PMC5214965; doi:10.1038/srep40022)
Supplement: Supplementary Information [file srep40022-s1.pdf]

Yersinia pestis halotolerance illuminates plague reservoirs

Maliya Alia Malek[1,2], Idir Bitam[1,2], Anthony Levasseur [1], Jérôme Terras[1], Jean Gaudart[1,3], Said Azza[1], Christophe Flaudrops[1], Catherine Robert[1], Didier Raoult[1], Michel Drancourt[1*]

1. Aix Marseille Université, URMITE, UMR 63, CNRS 7278, IRD 198, Inserm 1095, Faculté de Médecine, 27 Bd Jean MOULIN, 13385 Marseille Cedex 5, France

2. Laboratoire Biodiversité et Environnement : Interactions Génomes, Faculté des Sciences Biologiques Université des Sciences et de la Technologie Houari Boumediene, El Alia, Bab Ezzouar 16111, Algérie.

3. Aix-Marseille Université, UMR912 SESSTIM (INSERM/IRD/AMU), Faculté de Médecine, 27 Bd Jean Moulin, 13385 Marseille Cedex 5, France

Supporting Information file

Content:

Supplementary Figure 1.

Supplementary Table 1.

Supplementary Table 2.

Supplementary Table 3.

Supplementary Table 4.

Supplementary Figure 1. Co-localization of plague foci with salt lakes in North hemisphere. The figure was generated from the map of the software Google Maps/Google Earth and Google Maps/Google Earth APIs (https://www.google.com/permissions/geoguidelines.html).

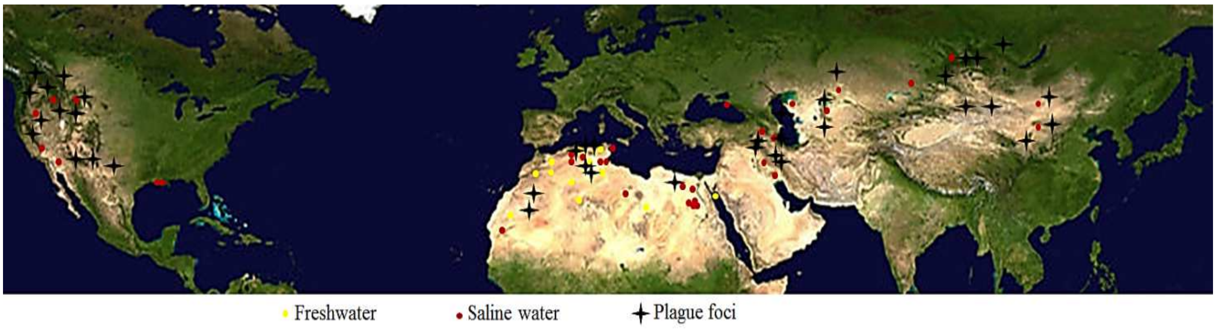

Supplementary Table 1. List of samples collected in North Algeria for the detection of *Y. pestis*.

Nature of samples	Area	Site of taking	pH	Salinity (g/L)	Effective	TOTAL
**Soil**	Oran	Sebkha (West side)	7.5 to 8	25 to 30	32	208
**Soil**	Oran	Sebkha (East side)	7.5 to 8	20 to 30	2	
**Soil**	Mostaganem	Bordjia	7.5	7	10	
**Soil**	Chelif	Chelif	7.5 to 8	14	8	
**Soil**	M'Sila	Chott El Hodna (Maarif)	7.5	70 to 140	20	
**Soil**	M'Sila	Chott El Hodna (Souamaa)	7.5	90 to 165	20	
**Soil**	M'Sila	Chott El Hodna (Ain el Khadra)	7.5	90 to 150	20	
**Soil**	M'Sila	Chott El Hodna burrow	7.5 to 8	15 to 45	36	
**Soil**	M'Sila	farm burrow	8	20	7	
**Soil**	M'Sila	farm burrow	7.5	8	3	
**Soil**	Biskra	burrow	7.5-8	15	15	
**Soil**	Biskra	farm	7.5	14	15	
**Soil**	Skikda	Guebli	7.5	2	10	
**Soil**	El Qala	El Mellah sand	7.5	17	10	
**Water**	Oran	Sebkha (Aïn Beida)	8	70 to 100	6	144
**Water**	Oran	Sebkha (Daët el Bagrat)	8	70 to 100	6	
**Water**	Oran	Sebkha (El Kerma)	8	70 to 100	6	
**Water**	Oran	Daiat Morsly	7.5	70	6	
**Water**	Oran	Daiat El Bagrat	7.5	65	6	
**Water**	Oran	Lake of Gharabas	7.5	25	6	
**Water**	Oran	Saline of Arzew	7.5	85	7	
**Water**	Mostaganem	Salt Lake (Bordjia)	7.5	40	5	
**Water**	Chelif	Wadi Chelif1	7.5	35	5	
**Water**	Chelif	Wadi Chelif2	7.5	40	5	
**Water**	Setif	Anoual	7.5	5	4	
**Water**	Setif	Djemila	7.5	1	4	
**Water**	Setif	Sebkha of Bazer	7.5	75	8	
**Water**	Biskra	Tolga	7.5	11	4	
**Water**	Biskra	Bouchegroune	7.5	1	4	
**Water**	Biskra	Ourelal	7.5	1	4	
**Water**	Biskra	Farfar	7.5	1	4	
**Water**	Biskra	Oumeche	7	10	4	
**Water**	Skikda	Oum Toub dam	7.5	2	4	
**Water**	Skikda	Saf-Saf wadi	7.5	3	4	
**Water**	Skikda	Guebli wadi	7.5	6	4	
**Water**	Annaba	Fetzara lake (North)	7.5	37	5	
**Water**	Annaba	Fetzara lake (East)	7.5	35	5	
**Water**	Annaba	Fetzara lake (South)	7.5	35	5	

**Water**	Annaba	Sea water	7.5	35	5	
**Water**	El Qala	El Mellah lake	8	37	7	
**Water**	El Qala	Oubeira lake	7.5	1	3	
**Water**	El Qala	Tonga lake	7.5	1	3	
**Water**	El Qala	Sea water	7.5	30	5	
TOTAL						352

Supplementary Table 2. Survival of *Y. pestis* Algeria 1 in a 40g/L salt after 5-week inoculation: number of colonies after 48-hour incubation on blood agar at 28°C, 5% CO_2

		**7 days**	**14 days**	**21 days**	**28 days**	**35 days**	**Mean**
Soil 1	1a	214	209	210	205	198	207.2
	1b	228	224	219	221	206	219.6
	1c	203	201	204	198	189	199
	Control 1	236	216	224	216	202	218.8
Soil 2	2a	232	228	219	224	213	223.2
	2b	221	216	214	217	211	215.2
	2c	229	209	213	213	207	214.2
	Control 2	219	212	215	210	197	210.6
Soil 3	3a	237	223	229	231	219	227.8
	3b	216	213	211	205	196	208.2
	3c	227	221	217	218	203	217.2
	Control 3	222	209	213	205	197	209.2

Supplementary Table 3. Identification of differentially expressed proteins between *Y. pestis* and *Y. pestis* exposed to 150g/L NaCl.

Spot number	Anova (p)	Fold Salt / WT	Protein name	Protein product	Locus tag	Mascot Score	MS Coverage
36	0,006496	1,900	L-lactate dehydrogenase	YP_002346582.1	lldD	145	38
21, 22	0,008875	1,600	bifunctional NADH:ubiquinone oxidoreductase subunit C/D	YP_002347514.1	nuoD	148	35
38, 39	0,015532	1,700	F0F1 ATP synthase subunit beta	YP_002348972.1	atpD	206	61
25, 26, 27	0,000251	1,700	F0F1 ATP synthase subunit alpha	YP_002348974.1	atpA	124	50
40, 41	0,027229	2,900	aspartate ammonia-lyase	YP_002345426.1	aspA	78	32
53	0,009047	1,700	glycine betaine/L-proline transport ATP-binding protein	YP_002347599.1	proV	69	33
20	0,032662	1,600	urease subunit alpha	YP_002347616.1	ureC	106	40
1	0,000179	2,900	phosphoenolpyruvate synthase	YP_002347374.1	ppSA	110	28
44, 45, 46	0,020615	3,200	maltoporin	YP_002348592.1	lamB	163	47
42, 43	0,029460	2,700	long-chain fatty acid outer membrane transporter	YP_002347691.1	fadL	179	62
86	0,000723	1,600	single-stranded DNA-binding protein, partial	YP_002345405.1	ssb	100	76
34	0,002340	1,900	replication-associated recombination protein A	YP_002346401.1	RarA	79	29
5	0,032218	2,000	outer membrane assembly complex, YaeT protein	YP_002346095.1	YaeT	123	32
54	0,000019	5,400	porin	YP_002346434.1	ompF	115	42
56, 58, 59, 60, 62	0,011032	2,100	outer membrane protein A	YP_002346456.1	ompA	235	72
35	0,014331	1,900	outer membrane channel protein	YP_002345731.1	tolC	151	46
68	0,005687	2,500	putative lipoprotein	YP_002346646.1	YPO1635	75	38
3	0,021044	1,600	hypothetical protein YPO1951	YP_002346940.1	hmsH	70	18
24	0,000548	1,600	hypothetical protein YPO2262	YP_002347229.1	YPO2262	117	44
47	0,037474	1,700	hypothetical protein YPO3839	YP_002348717.1	YPO3839	91	42
48	0,019104	0,556	succinyl-CoA synthetase subunit beta	YP_002346158.1	sucC	114	40
28, 29	0,001039	0,625	dihydrolipoamide dehydrogenase	YP_002348309.1	lpdA	137	50
52	0,007958	0,417	isocitrate lyase	YP_002348605.1	aceA	179	57
85	0,009023	0,323	F0F1 ATP synthase subunit delta	YP_002348975.1	atpH	123	61

57	0,000311	0,455	D-lactate dehydrogenase	YP_002347296.1	hslI	102	48
10	0,000101	0,294	biodegradative arginine decarboxylase	YP_002346237.1	adiA	39	11
33	0,000417	0,345	multifunctional aminopeptidase A	YP_002348332.1	pepA	67	27
64	0,015560	0,625	uridine phosphorylase	YP_002348663.1	udp	83	45
49, 50, 51	0,001512	0,667	enolase	YP_002348268.1	eno	78	36
65, 66, 67	0,002091	0,588	short chain dehydrogenase	YP_002348103.1	fabG2	74	37
101	0,003968	0,435	50S ribosomal protein L9	YP_002348425.1	rplI	79	36
23	0,009187	0,556	transcription elongation factor NusA	YP_002348386.1	nusA	178	47
61, 63, 78	0,000604	0,455	outer membrane protein A	YP_002346456.1	ompA	82	38
94	0,003142	0,625	peptidyl-prolyl cis-trans isomerase B (rotamase B)	YP_002347994.1	ppiB	125	77
9	0,044262	0,588	hsp90 family protein, partial	YP_002348027.1	htpG	44	20
87, 88, 89	0,003240	0,588	putative alkyl hydroperoxide reductase subunit c	YP_002348099.1	ahpC	76	36
31	0,014587	0,625	catalase	YP_002346241.1	katE	140	33
95	0,011068	0,345	DNA starvation/stationary phase protection protein Dps	YP_002347474.1	dps	126	65
16, 17, 18, 19	0,000029	0,270	catalase/peroxidase HPI, partial	YP_002348215.1	katY	130	38
55	0,012553	0,625	nucleoid-associated protein NdpA	YP_002346291.1	NdpA	105	63
91, 92, 93	0,022952	0,476	hypothetical protein YPO0502	YP_002345575.1	YPO0502	110	62
98	0,008342	0,270	universal stress global response regulator UspA	YP_002348841.1	uspA	64	37

Supplementary Table 4. Primers and probes used for PCR.

Plasmid	Target (nucleotide sequence accession number)	Primer or probe sequence (5'→3 ')		Size (bp) of PCR product
pPla	*pla* (M27820)	YpPla F20	ATGGAGCTTATACCGGAAAC	98
		YpPla R18	GCGATACTGGCCTGCAAG	
		YpPla S	6FAM-TCCCGAAAGGAGTGCGGGTAATAGG-TAMRA	
pMT1	*caf1* (AF053945)	Ypcaf F	TACGGTTACGGTTACAGCAT	240
		Ypcaf R	GGTGATCCCATGTACTTAACA	
	ymt (X92727)	Yptox F	AGGACCTAATATGGAGCAATGAC	168
		Yptox R	CTAACAAAGCCTCAATAATCCA	
pCD1	*yopT* (AL117189)	Yop F	GATCAGGAGCCATGCCACAA	330
		Yop R	ACATTTGGCCTGAGAGATGTA	
